# Poly(styrene-*ran*-cinnamic acid) (SCA), an approach to modified polystyrene with enhanced impact toughness, heat resistance and melt strength

**DOI:** 10.1039/c9ra08635h

**Published:** 2019-12-02

**Authors:** Jie Wang, Zixin Yu, Peihua Li, Dachuan Ding, Xuan Zheng, Chuanqun Hu, Tao Hu, Xinghou Gong, Ying Chang, Chonggang Wu

**Affiliations:** Hubei Provincial Key Laboratory of Green Materials for Light Industry, Collaborative Innovation Centre of Green Light-weight Materials and Processing, School of Materials and Chemical Engineering, Hubei University of Technology Wuhan Hubei 430068 P. R. China cgwu@mail.hbut.edu.cn

## Abstract

A poly(styrene-*ran*-cinnamic acid) (SCA) containing 6.8 mol% of CA, with a *M̄*_w_ (∼217 000) comparable to commercial polystyrene (PS), was successfully synthesised *via* emulsion free-radical copolymerisation as evidenced by 1744 and 1703 cm^−1^ infrared peak occurrences, respectively characteristic of free and dimeric carboxyl C=O stretches. Upon the interchain hydrogen bond cross-linking by CA, the impact toughness of the SCA was considerably improved by 47.2% against PS, the glass transition, heat deflection and Vicat softening temperatures were significantly enhanced until 117.0, 108.0 and 118.3 °C, respectively, compared with PS (95.2, 87.6 and 96.0 °C), while the extensional viscosities were near one order-of-magnitude higher than PS by which the temperature window required for appropriate melt-strengths would be greatly broadened. Meanwhile, the SCA displayed other properties basically analogous to PS. This work presents a modified PS, SCA, with enhanced toughness, heat resistance and melt strength that potentially extend its styrofoam and commodity applications.

## Abstract 2

Carboxyl-bearing cinnamic-acid copolymerised polystyrene exhibits enhanced impact toughness, heat resistance and melt strength due to its reversible hydrogen bond cross-linking.

## Graphical abstract

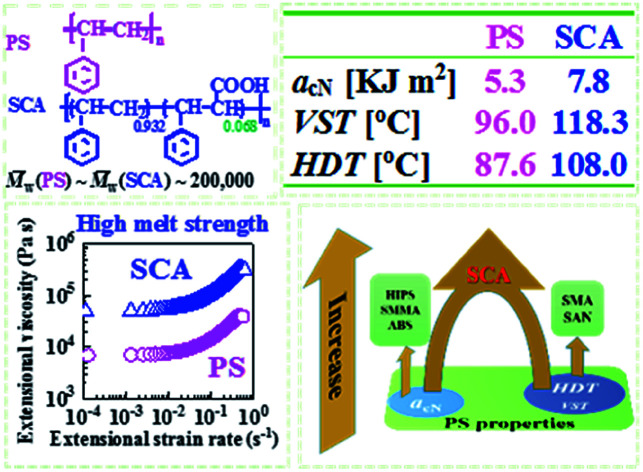

## Introduction

Since its commercialisation, polystyrene (PS), a commodity resin second in consumption only to polyethylene and poly(vinyl chloride) (PVC), has widely been used in packaging materials, automotive interiors, electronic components, *etc*. because of its good transparency, high modulus, superior electrical insulation, satisfactory dyeability, low price and excellent processability. Nevertheless, owing to the presence of bulky, rigid pendant phenyl groups as well as weak, nonpolar chain-segmental interactions, PS exhibits poor (impact) toughness, *i.e.* high brittleness; PS does not have high enough heat resistance as well to extend itself to engineering applications, and more importantly, the low melt-strength of PS limits its foamability during styrofoam production. To overcome these disadvantages especially high brittleness, researchers have made a number of modifications to improve the toughness or heat resistance of PS. Some melt-blended elastomers, *e.g.* natural rubber,^1^ butadiene rubber (BR),^2,3^ ethylene–propylene–(diene monomer) rubber,^4^*etc.*, into PS matrices to enhance the toughness at the sacrifice of the heat resistance of PS, among which the BR-modified PS constituted commercialised high-impact polystyrene (HIPS); meanwhile, rigid polymers, *e.g.* poly(2,6-dimethyl-1,4-phenylene oxide),^5^ are blended with PS to enhance its heat resistance as well as mechanical strength, however, with its toughness reduced. Ceramic fillers, *e.g.* nano-CaCO_3_,^6^ nano-Ca_3_(PO_4_)_2_,^7^ functionalised graphene oxide,^8^ SiO_2_,^9^ expanded graphite,^10^ TiO_2_,^11^*etc.*, also fill PS to raise its heat resistance and/or mechanical strength, while its toughness is not elevated; even worse, most of the fillers cause serious wear and tear upon processing equipment such as screw extruders. (Covalent) cross-linking modification^12,13^ is used as well to increase the heat resistance and mechanical strength of PS, which, nevertheless, is only applicable to PS products instead of resins. Others modified PS by copolymerisation to increase its heat resistance and/or mechanical strength (*e.g.* commercial styrene-maleic anhydride (SMA)^14–16^ and styrene-acrylonitrile (SAN),^17,18^ styrene-*N*-phenyl maleimide,^19^*etc.*) or to enhance its toughness (*e.g.* styrene–methyl methacrylate (SMMA),^1,20–22^ acrylonitrile–butadiene–styrene (ABS),^23,24^*etc.*), whereas nearly all of the comonomers reported in the literature are hazardous or harmful to the environment. Obviously, although people's PS-modification endeavours have given rise to commercial styrenics like HIPS, SMA, SAN, SMMA and ABS, few modifications improve both the toughness and heat resistance of PS.

Herein, an innovative modification of PS is made by copolymerising styrene with a fully green monomer, cinnamic acid (CA), recommended as a “generally recognised as safe” (GRAS) compound by the U.S. Food and Drug Administration,^25^ for improving both its toughness and heat resistance, as well as melt strength, by the introduction of reversible hydrogen bond cross-links.^26^ First, the molecular weight (*M̄*_w_) of the product, supposedly a poly(styrene-*ran*-cinnamic acid) (SCA), is tailored to a commercial grade of 200–250k by adjustment of the process conditions of the emulsion free-radical copolymerisation. Then, the chemical structure and CA content, respectively, of the SCA are verified by infrared spectroscopy and acid–base titration. Subsequently, as a styrenic, the SCA is systematically characterised in terms of its mechanical, thermal, processing, dielectric, fire and other physical properties, in which an emphasis is placed on the examination of its toughness, heat resistance and melt strength.

## Experimental

### Materials

Styrene (≥98.0%), NaOH (≥96.0%), ethanol, anhydrous (≥99.7%), Al_2_(SO_4_)_3_·18H_2_O (≥99.0%), toluene (≥99.5%), KOH (≥85.0%), all of analytical reagent, and phenolphthalein (indicator grade) were purchased from Sinopharm (Shanghai) Chemical Reagents Co., Ltd., China. Na_2_SO_4_, anhydrous (≥99.0%) and sodium dodecylsulfate (SDS), both of analytical purity, were supplied by Tianjin City Fuchen Chemical Reagents Plant, China. Potassium persulfate (K_2_S_2_O_8_) (analytical purity, ≥99.5%) was obtained from Younaide Initiators (Shanghai) Co., Ltd., China. (*Trans*-)CA (analytical reagent, 99.0%) was provided by Shanghai Macklin Biochemical Co., Ltd., China. A PS resin (injection grade, GPPS 123P) was received from Shanghai Secco Petrochemical Co., Ltd., China. Tetrahydrofuran (THF) (standard for GC, >99.9%) and KBr (spectral reagent, 99.0%) were offered by Aladdin Industrial Corp., China. Potassium biphthalate (working chemical, ≥99.9%) was afforded by Tianjin City Chemical Reagents Institute Co., Ltd., China. Deionised water was home made in our laboratory using an ultra aqua pura machine (Ulupure, China, UPT-I-10T).

Prior to its use, styrene was purified by extractions with a 5 wt% of NaOH aqueous solution to remove polymerisation inhibitor(s) and impurities and then with deionised water to eliminate the residual NaOH, followed by drying with anhydrous Na_2_SO_4_. K_2_S_2_O_8_ was also refined by recrystallisation in a 0 °C ice-water bath from 50 °C deionised water, followed by filtration, washing with ice water and then with anhydrous ethanol repeatedly, air-drying in a fume hood overnight and finally vacuum drying at 50 °C for at least 12 h. All the other chemicals were used as received without any further purification unless otherwise specified below.

### Emulsion free-radical copolymerisation of styrene and cinnamic acid

Styrene was copolymerised with a small amount (10 wt%) of CA using an emulsion method. An overhead stirrer as well as a laboratory condenser was fitted to a 2000 mL three-necked round-bottom flask clamped and immersed in a 50 °C water bath, into which deionised water (600 mL) and SDS emulsifier (∼0.8080 g) were added. Upon 250 rpm stirred emulsification of the mixture for 0.5 h, the (purified) styrene monomer (60 mL) and CA comonomer (20.20 g) were added to the flask, after which the water-bath temperature was increased to 75 °C; meantime, the (refined) K_2_S_2_O_8_ (3.2320 g) was dissolved in deionised water (75 mL) in a 100 mL beaker to prepare an initiator solution. While the bath temperature was stabilised at 75 °C, the initiator solution (25 mL) was poured into the stirred reaction system. Once the reaction mixture appeared bluish or little liquid was observed to reflux, the bath temperature further was increased to 80 °C, when the styrene (70 mL) and initiator solution (25 mL) were sequentially decanted into the system. On their constant-temperature reaction for 1 h, the remaining styrene (70 mL) and initiator solution (25 mL) were successively dumped in, which were then reacted, again, at 80 °C for 1 h. At this juncture, the system was allowed to react for another 0.5 h at an enhanced temperature of 85 °C, contingent upon whether there was noticeably refluxed liquid (*i.e.* styrene) observed. After the copolymerisation reaction was completed, the emulsion was transferred to a 1000 mL beaker and cooled to room temperature (RT), into which Al_2_(SO_4_)_3_ demulsifier (∼1.6 g) was then poured to break the emulsion under vigorous stirring. The obtained white product was Büchner filtrated, then transferred to a 1000 mL beaker and subsequently soaked in hot (70–80 °C) deionised water (200–300 mL) for 5 min under stirring; such a process was repeated at least 3 times. Afterwards, a similar process was repeated at least 3 times with RT anhydrous ethanol as the soaking solvent. Finally, the product was Büchner filtrated, then air-dried in a fume hood for at least 3 days and subsequently vacuum dried at 90 °C for at least 1 day to supposedly obtain an SCA resin.

### Characterisations of the SCA

#### Gel permeation chromatography (GPC)

To ascertain if it had a comparable *M̄*_w_ of 200–250k to the (commercial) PS resin, the (potential SCA) product resin was measured at 35 °C against the PS using a gel permeation chromatograph (Waters, Waters 1525). For the runs, THF was used as the eluent at a constant flow rate of 1.0 mL min^−1^ and monodisperse molecular weights (up to 1200k) of PS resins as the standards.

#### Fourier transform infrared (FTIR) spectroscopy

To verify success in the SCA synthesis, the FTIR spectrum of the product resin was analysed against that of the PS in a mid-IR range of 4000–500 cm^−1^. An FTIR spectrometer (Bruker, Tensor II) was employed to collect the absorption spectra of a 4 cm^−1^ resolution at RT based on 24 scans in the transmission mode. The samples were prepared as KBr pellets by grinding together, using a pestle and mortar, either of the two polymer powders (∼2.0 mg) and the KBr (∼50 mg) dried with an infrared baking lamp, followed by RT compression of the mixture powder on a hydraulic press. Prior to the samples preparation, both the product resin and PS were purified by reprecipitation from toluene into a large amount of anhydrous ethanol, followed by Büchner filtration, washing with anhydrous ethanol repeatedly, air-drying in a fume hood overnight and finally vacuum drying at 90 °C for at least 12 h.

#### Acid–base titration

A toluene solution (∼3.3 × 10^−3^ g mL^−1^) of an accurately weighed mass of the well-dried, purified SCA was titrated in an Erlenmeyer flask against a KOH ethanol solution from a basic burette, which, with a nominal concentration of 1.0 × 10^−2^ M, was in turn titrated *in situ* to evaluate its effective concentration with a precise amount of potassium-biphthalate standard of ∼1.0 × 10^−3^ g mL^−1^ in ethanol in another Erlenmeyer flask. For both the titrations, a phenolphthalein indicator solution in an ethanol/water (19/1 v/v) mixed solvent (∼1.0 × 10^−2^ g mL^−1^) was used to identify the first occurrence of a reddish colour as the equivalence point. The CA content (mol%), *c*_CA_, of the SCA was therefore calculated using the following eqn (1), 1
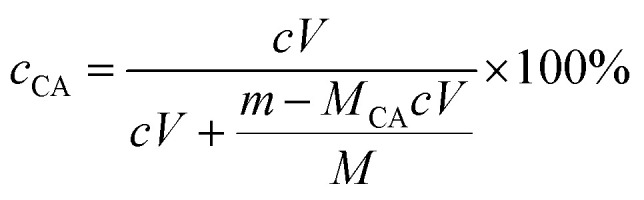
where *c* is the effective molar concentration (mol L^−1^ or M) of the KOH ethanol solution, *V* the volume (L) of the KOH ethanol solution consumed until the equivalence point of the SCA toluene solution titration, *m* the accurately weighed mass (g) of the well-dried SCA, *M* and *M*_CA_ the molar masses (g mol^−1^) of styrene and CA units (*i.e.* 104.15 and 148.16), respectively. To minimise any uncertainty of the result, the median *c*_CA_ value from five parallel titrations was taken as the *c*_CA_ of the SCA synthesised.

#### Mechanical testing

The well-dried SCA and PS resins either were plasticated at 200 °C in a plasticorder (Harbin Harp Electrical Technology Co., Ltd., China, RM-200C) for 6 min at a screw speed of 80 rpm, and then ground into a powder with a laboratory grinder (Yongkang City Boou Hardware Factory, China, 400Y). A laboratory mini injection molder (Shanghai Xinshuo Precision Machinery Co., Ltd., China, MiniJet WZS10D) was subsequently used to mold standard impact, tensile and flexural specimens, with the barrel and mold temperatures of 220 °C (SCA)/200 °C (PS) and 60 °C, respectively. Finally, the notched impact strength was tested using a Charpy impact tester (Chengde City Testing Machinery Co., Ltd., China, XJJ-50) according to the ISO 179-1 testing standard (specimen type 1). The tensile properties were measured on a universal testing machine (MTS (China), CMT-4202) in the light of the ISO 527 testing standard, using ∼2 mm thick dumbbell-shaped specimens with a gauge length of 20 mm and a crosshead speed of 2 mm min^−1^; in each of the stress–strain curves, the fracture strength was defined as the tensile strength since none of them exhibited a yield behaviour. The flexural properties were measured on the same machine in accordance with the ISO 178 testing standard, with rectangular specimen dimensions of 80 × 10 × 4 mm and a loading-edge speed of 2 mm min^−1^.

#### Differential scanning calorimetry (DSC)

The glass transition temperature (*T*_g_) of the SCA resin was measured against the PS resin according to the ISO 11357 testing standard using a differential scanning calorimeter (PerkinElmer, DSC 8000) under a N_2_ atmosphere of 20 mL min^−1^, upon its calibrations in both temperature and heat flow with an indium standard. For the runs, the well-dried samples (∼3.5 mg), encapsulated into a flat-bottomed aluminum pan, were enclosed in the DSC sample cell while an empty pan–lid capsule in the reference cell. Both the cells were then heated from 30 to 200 °C to minimise any effect(s) of sample preparation history, followed by cooling to 30 °C and finally heating again to 200 °C, all at a rate of 20 °C min^−1^. Prior to the sample runs, a baseline was acquired by running the same heating–cooling–heating consecutive cycles of the cells both enclosing an empty pan–lid capsule. Therefore, in the second heating cycle, sample-mass-normalised, baseline-subtracted heat flow (W g^−1^) was plotted against temperature (°C) to constitute the DSC thermograms of the resins for analysis. In either thermogram, the inflection point (°C) of the glass-transition step was identified as the *T*_g_.

#### Heat deflection temperature (HDT) testing

The well-dried SCA and PS resins either were plasticated, then ground, and subsequently injection-molded into standard rectangular (80 × 10 × 4 mm) specimens using the same procedure as for the flexural testing. The HDT values of the specimens were finally measured in accordance with the ISO 75 testing standard using a heat deflection and Vicat softening temperatures tester (Shenzhen City Aode Saichuang Technology Co., Ltd., China, Auto-RBWK), during their heating in a methyl-silicone oil bath from 25 to 200 °C at a rate of 120 °C h^−1^.

#### Vicat softening temperature (VST) testing

An 80 × 10 × 4 mm rectangle of the SCA and PS resins, prepared using the same procedure as for the flexural testing, was cut into three 10 × 10 × 4 mm specimens for VST measurement according to the ISO 306 testing standard (method A50). For that purpose, the same tester as for the HDT testing was used to measure the specimens during their heating from 25 to 200 °C.

#### Thermogravimetric analysis (TGA)

The thermal decomposition behaviour of the well-dried SCA resin was studied against the PS resin in accordance with the ASTM E2402 testing standard using a thermogravimetric-differential-thermal simultaneous analyser (TA Instruments, SDT Q600). A small amount (∼3.0 mg) of either of the resins was placed in a disposable Al_2_O_3_ crucible on one tray of the thermal balance, which, along with an empty Al_2_O_3_ crucible on the other tray, was then heated from 30 to 600 °C at a rate of 20 °C min^−1^ under a N_2_ atmosphere of 100 mL min^−1^. The temperature at which a 5% weight loss of the sample occurred was defined as its thermal decomposition temperature.

#### Linear thermal expansion coefficient measurement

The measurement was performed in the light of the ASTM E831 testing standard. For that purpose, the SCA and PS resins were compression-molded at 220 °C (SCA)/200 °C (PS) and 15 MPa into a 100 × 90 × 1 mm rectangle, which was then cut into three 2 × 1 × 1 mm specimens. A static thermomechanical analyser (TA Instruments-Waters LIC, TMA Q400EM) was subsequently used to measure the length in the thickness direction of the specimens as a function of temperature during their heating from 20 to 80 °C at a rate of 5 °C min^−1^, from which the coefficient of linear thermal expansion, *α*_m_, was finally calculated.

#### Thermal conductivity measurement

100 × 90 × 1 mm rectangles of either of the SCA and PS resins, prepared using the same procedure as for the *α*_m_ measurement, were cut into three specimens of 40 × 40 × 1 mm, the thermal conductivity of which was then measured with a portable thermal conductivity meter (Xi'an City Xiaxi Electronic Technology Co., Ltd., China, TC 3000E) according to the ASTM D5930 testing standard.

#### Melt strength measurement

The extensional rheological properties of the SCA resin were measured at 220 °C against the PS resin at 200 °C using an extensional rheometer (Göttfert, Rheotens 71.97), continuously fed by a high-pressure capillary rheometer (Göttfert, Rheograph 50) with plunger diameter and speed of 15 mm and 0.4 mm s^−1^, and die diameter and aspect-ratio of 2 mm and 10, respectively. Accordingly, the extrusion rate of the melt at the die exit was 11.3 mm s^−1^; in the extensional rheometer, the nip rollers were counter-rotated with a roller spacing of 0.4 mm and a linear acceleration of 12 mm s^−2^; the gauge length (*i.e.* distance between the die exit and the symmetrical axis between the rollers) was set at l00 mm.

#### Melt flow rate (MFR) testing

A melt index tester (Chengde City Jinjian Testing Instruments Co., Ltd., China, MFI 1211) was used to measure the MFR of the SCA and PS resins according to the ISO 1133 testing standard, with load and temperature of 5.0 kg and 200 °C, respectively.

#### Shear rheometry

The complex viscosity magnitude (|η*|) of the SCA and PS resins was measured at 200 °C under a N_2_ atmosphere as a function of angular frequency (*ω*) (10^−2^–3.8 × 10^2^ rad s^−1^) in the oscillatory shear mode using a rotational rheometer (TA Instruments, DHR-2), with a parallel-plate fixture of 25 mm in diameter and 1.0 mm in separation. To ensure linear viscoelastic behaviour of the resin melts, a strain-amplitude (*ε*) sweep experiment was conducted at 200 °C and 60 Hz (*i.e.* 3.8 × 10^2^ rad s^−1^) prior to the measurements to determine its safe value to be applied. Before the test, amounts (∼1.4 g) of either resin were compression-molded at 220 °C (SCA)/200 °C (PS) and 15 MPa into discoid specimens of ∼25 mm in diameter and ∼2 mm in thickness. On the other hand, the shear viscosities (*η*'s), at higher shear rates (*γ̇*’s) (∼10^2^–10^4^ s^−1^), of the resins were measured at 200 °C by a twin-bore capillary rheometer (Malvern Panalytical, Rosand RH10) with one die aspect-ratio (*L*/*D* = 10/1) against the other (*L*/*D* = 0.17/1) for the Bagley correction. The acquired raw data were analysed by the Rabinowitch correction to obtain the flow-curve results.

#### Dielectric properties measurement

Discoid (*∅* 25 × 2 mm) specimens, prepared following the same procedure as for the oscillatory-shear specimens, were used to measure the dielectric-constant (*ε*) and dielectric-loss (tan *δ*) of the SCA and PS resins according to the IEC 60250 testing standard. The resonance voltages and resonant capacitors were measured with and without the specimens at 60 Hz and 1 MHz to evaluate the *ε*'s and tan *δ*'s, using an LCR digital bridge (Shanghai Tongbei Testing Technologies Co., Ltd., China, TH2826).

#### Oxygen index testing

Twenty 80 × 10 × 4 mm rectangular specimens, prepared using the same procedure as for the flexural testing, were employed to measure the oxygen index of the SCA and PS resins at RT (23 ± 2 °C) with an oxygen index tester (Nanjing City Jiangning Analytical Instruments Co., Ltd., China, JF-3) in accordance with the ISO 4589 testing standard. Prior to the test, the equipment was recalibrated, and, during the test, the glass chimney was cleaned constantly to maintain good visibility.

#### Smoke density testing

The smoke-density-rating value of the SCA was measured against that of the PS using a smoke density tester (Nanjing City Jiangning Analytical Instruments Co., Ltd., China, JCY-2) in the light of the ASTM D2843 testing standard. For the test, an amount (∼30 g) of either resin was compression-molded at 220 °C (SCA)/200 °C (PS) and 15 MPa into a 60 × 60 × 6 mm rectangle, which was then cut into three 25 × 25 × 6 mm specimens.

#### Intrinsic viscosity measurement

Following the ISO 1682-1 testing standard, an Ubbelohde viscometer was used to measure the intrinsic viscosity of the SCA relative to the PS. For the runs, either of the resins was dissolved at 25 ± 0.1 °C in toluene to prepare solutions of five decreasing concentrations, *i.e.* 6/6, 5/6, 2/3, 1/2 and 2/5 of 0.01 g mL^−1^.

#### Specific gravity measurement

Five *∅* 25 × 2 mm discoid specimens, prepared following the same procedure as for the oscillatory-shear specimens, were used to measure the specific gravity of the SCA and PS resins with a solid density meter (Beijing City Etnaln Electronic Technologies Co., Ltd., China, ET-320) according to the ASTM D792 testing standard.

#### Water absorption measurement

100 × 90 × 1 mm rectangles of either of the SCA and PS resins, prepared using the same procedure as for the *α*_m_ measurement, were cut into three 60 × 60 × 1 mm specimens, the water absorption of which was then measured upon their immersion in deionised water at 23 ± 1 °C for 24 ± 1 h, in accordance with the ISO 62 testing standard.

To ensure statistical significance, the medians from five parallel results were taken as the data for analysis for the titration, dielectric-property and specific-gravity measurements, from at least eight results for the mechanical-property and MFR tests and from three results for the HDT, VST, *α*_m_, thermal-conductivity, smoke-density and water-absorption measurements.

## Results and discussion

### Reproducibility of the emulsion free-radical copolymerisation

In Table 1, the (potential SCA) product resin (Table 1(A)) synthesised had molecular weights (*M̄*_n_, *M̄*_w_, *M*_p_, *M̄*_z_ and *M̄*_z+1_) and their distributions (*D*_1_, *D*_2_ and *D*_3_) all comparable to those of the (commercial) PS. More synthetic batches (Table 1(B–F)) of the product resin were all not changed considerably in molecular weights and their distributions compared with Table 1(A), indicating that the polymerisation process was controlled at high reproducibility.

**Table 1:** Molecular weights and their distributions, measured from six parallel batches of polymerisations, of supposedly a poly(styrene-*ran*-cinnamic acid) (SCA), *i.e.* polystyrene (PS) copolymerised with a small amount of cinnamic acid, compared with those of a commercial PS

Resin	Polymerisation no.	*M̄*_n_	*M̄*_w_	*M*_p_	*M̄*_z_	*M̄*_z+1_	*M̄*_w_/*M̄*_n_(*D*_1_)	*M̄*_z_/*M̄*_w_(*D*_2_)	*M̄*_z+1_/*M̄*_w_(*D*_3_)
PS	—a	60 000	198 000	151 000	414 000	611 000	3.3	2.1	3.1
SCA	A	60 000	217 000	157 000	467 000	685 000	3.6	2.2	3.2
	B	64 000	209 000	155 000	439 000	656 000	3.3	2.1	3.1
	C	30 000	224 000	188 000	483 000	698 000	7.2	2.2	3.1
	D	30 000	210 000	174 000	463 000	681 000	6.9	2.2	3.3
	E	60 000	227 000	176 000	474 000	690 000	3.8	2.1	3.0
	F	38 000	227 000	188 000	486 000	704 000	6.0	2.1	3.1

a Not applicable.

### Evidence for the successful synthesis of SCA

**Fig. 1:**
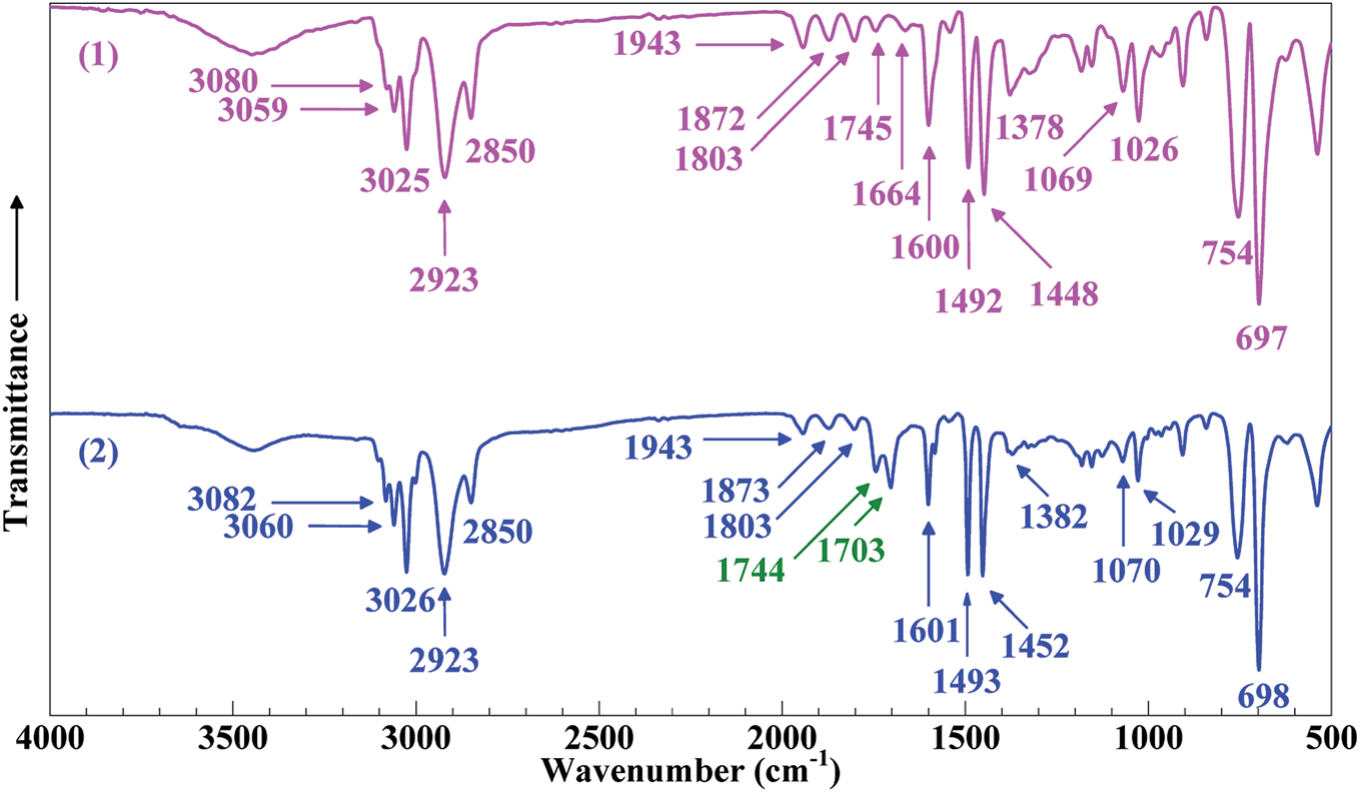
Fourier transform infrared (FTIR) absorption spectra of (1) a commercial polystyrene (PS) and (2) a resin, supposedly poly(styrene-*ran*-cinnamic acid), laboratory synthesised by emulsion free-radical copolymerisation of styrene with a small amount (10 wt%) of cinnamic acid.

Shown in Fig. 1 is the FTIR absorption spectrum (Trace 2) of the product resin as opposed to that (Trace 1) of the PS. In Trace 1 for the PS, the absorption bands at 3080, 3059 and 3025 cm^−1^ were attributed to the stretches of the phenyl C–H bonds,^27^ the 2923 and 2850 cm^−1^ bands, respectively, to the antisymmetric and symmetric stretches of the –CH_2_– groups,^28^ the 1943, 1872, 1803, 1745 and 1664 cm^−1^ bands to the C–C and C–H overtone and combination stretches of the phenyl groups,^29^ the 1600, 1492 and 1448 cm^−1^ absorption peaks to the stretches of the phenyl C–C bonds (*i.e.* phenyl skeleton vibrations),^27,30^ the 1378 cm^−1^ peak to the in-plane rocking of the aliphatic CH groups in the main chain,^29^ the 1069 and 1026 cm^−1^ and 754 and 697 cm^−1^ peaks, respectively, to the in-plane and out-of-plane bending of the phenyl C–H bonds.^31^ Compared with Trace 1, Trace 2 for the product resin exhibited, in addition to all the above featured absorptions, new peaks at 1744 and 1703 cm^−1^ characteristic of the stretches of free and dimeric carboxyl C=O bonds, respectively.^13,32^ Right around there, part of the phenyl overtone and combination stretches (at 1745 and 1664 cm^−1^) were found to be overshadowed due to their weaker intensities, which does not prevent one inferring that the 1744 and 1703 cm^−1^ bands were indeed new from the introduced carboxyls.

Contingent upon the reactivity ratios of styrene and CA monomers, the product resin might primarily comprise one of three possible species, PS-poly(cinnamic acid) (PCA) blend, PS and SCA. From the resin's FTIR sample reprecipitated from toluene into ethanol (*cf.* Par. 2, Section 2.3), free CA and/or PCA molecules, if any, were effectively eliminated by the dissolving ethanol. Therefore, if it had been a PS–PCA blend or PS, the resin would have demonstrated an FTIR spectrum basically of neat PS (Trace 1, Fig. 1), which was impossible. In other words, upon the removal of any free (*i.e.* unpolymerised) CA from it, the resin actually displayed an FTIR spectrum (Trace 2, Fig. 1) bearing the carboxyls' characteristic bands, which proves success in the copolymerisation of CA into PS to form an SCA. More quantitatively, the CA content of the SCA was evaluated from eqn (1) by acid–base titration to be 6.8 mol%, which was indeed significant considering the CA-comonomer feed ratio of 10 wt% (∼7.2 mol%). This value, along with the SCA's typical *M̄*_w_ and *M̄*_w_/*M̄*_n_ against those of the PS (Table 1), is listed in Table 2.

**Table 2:** Overall properties of a poly(styrene-*ran*-cinnamic acid) (SCA) resin, *i.e.* polystyrene (PS) copolymerised with 6.8 mol% of cinnamic acid, compared with those of a commercial PS resin

Properties	Parameter	Testing method	Testing condition(s)	Value	
				PS	SCA
Structural	Cinnamic-acid (CA) content (mol%)	—a	—a	—c	6.8
	Weight-average molecular weight (*M̄*_w_)	—b	35 °C	198 000	217 000
	Polydispersity index (*M̄*_w_/*M̄*_n_)	—b	35 °C	3.3	3.6
Mechanical	Charpy notched impact strength (kJ m^−2^)	ISO 179-1	23 ± 2 °C, 2 mm min^−1^	5.3	7.8
	Young's modulus (MPa)	ISO 527	23 ± 2 °C, 2 mm min^−1^	2.8 × 10^2^	3.7 × 10^2^
	Tensile strength (MPa)	ISO 527	23 ± 2 °C, 2 mm min^−1^	52.3	57.1
	Elongation at break (%)	ISO 527	23 ± 2 °C, 2 mm min^−1^	10.5	9.7
	Flexural modulus (MPa)	ISO 178	23 ± 2 °C, 2 mm min^−1^	2.1 × 10^4^	2.4 × 10^4^
	Flexural strength (MPa)	ISO 178	23 ± 2 °C, 2 mm min^−1^	138.4	167.6
Thermal	Glass transition temperature (°C)	ISO 11357	20 °C min^−1^	95.2	117.0
	Heat deflection temperature (°C)	ISO 75	0.45 MPa, 120 °C h^−1^	87.6	108.0
	Vicat softening temperature (°C)	ASTM D1525	10 N, 50 °C h^−1^	96.0	118.3
	Thermal decomposition temperature (°C)	ASTM E2402	20 °C min^−1^	391.9^d^	360.1^d^
	Linear thermal expansion coefficient (μm m^−1^ °C^−1^)	ASTM E831	25–75 °C, 5 °C min^−1^	78.4	79.5
	Thermal conductivity (W m^−1^ K^−1^)	ASTM D5930	23 ± 0.1 °C	0.160	0.165
Processing	Melt strength^e^ (Pa s)	—f	0.5 s^−1^, 220 °C (SCA)/200 °C (PS)	4.2 × 10^4^	3.3 × 10^5^
	Melt flow rate (g per 10 min)	ISO 01133	200 °C, 5.0 kg	6.9	0.6
	Shear viscosity (Pa s)	—g	200 °C, 10^3^ s^−1^	101.7	278.9
Dielectric	Dielectric constant	IEC 60250	60 Hz	2.8	2.8
			1 MHz	2.8	2.7
	Dielectric loss	IEC 60250	60 Hz	3.7 × 10^−3^	3.3 × 10^−3^
			1 MHz	0.6 × 10^−3^	1.6 × 10^−3^
Fire	Oxygen index (vol%)	ISO 4589	23 ± 2 °C	19.7	19.9
	Density of smoke (%)	ASTM D2843	23 ± 2 °C	88.8	92.5
Other physical	Intrinsic viscosity (dL g^−1^)	ISO 1628-1	25 ± 0.1 °C, toluene	8.4 × 10^−2^	2.6 × 10^−2^
	Specific gravity	ASTM D792	23 ± 2 °C	1.05	1.07
	Water absorption (wt%)	ISO 62	23 ± 2 °C, 24 h	0.06	0.47

a Measured by titration of a toluene solution of the purified SCA against a KOH ethanol solution titrated by potassium-biphthalate standard, with a phenolphthalein solution in an ethanol/water (19/1 v/v) mixed solvent as the indicator.

b Measured at 35 °C by gel permeation chromatography (GPC), with tetrahydrofuran as the eluent at a constant flow rate of 1.0 mL min^−1^ and monodisperse PS resins up to 1200k as the standards.

c Not applicable.

d Measured by thermogravimetric analysis (TGA) at a heating rate of 20 °C min^−1^ under a N_2_ atmosphere, with the temperature at 5% weight loss defined as the *T*_d_.

e Characterised by the extensional viscosity (*η*_e_) at a practical extensional strain rate (*ε̇*) of 0.5 s^−1^ in the *η*_e_*vs. ε̇* flow curves.

f Measured at 200 °C (PS)/220 °C (SCA) by an extensional rheometer, with a die-exit melt extrusion rate of 11.3 mm s^−1^, a nip-rollers’ linear acceleration of 12 mm s^−2^, and a gauge length (*i.e*. distance between the die exit and the nip rollers) of 100 mm.

g Measured at 200 °C by a twin-bore capillary rheometer with one die aspect-ratio (*L*/*D* = 10/1) against the other (*L*/*D* = 0.17/1) for the Bagley correction.

### Mechanical properties of the SCA

As shown in Table 2, the Charpy notched impact strength, tensile and flexural properties (primarily modulus and strength) of the SCA were all enhanced compared with the (commercial) PS, which may arise from, besides the volume fraction (6.8 mol%) of rigid CA comonomer, essentially the reversible hydrogen bond cross-links (*i.e.* dimeric carboxyls) introduced by the copolymerisation of CA. Particularly worth noting is that the impact toughness of the SCA was significantly improved by 47.2% against the PS. According to the craze theory,^33,34^ this might be associated with balanced initiation of new crazes by unzipping (*i.e.* de-hydrogen-bonding) of SCA segments and stabilisation of preexisting crazes propagation by segmental zipping (*i.e.* re-hydrogen-bonding), both of which contributed to absorption of impact energy.

### Thermal properties of the SCA

**Fig. 2:**
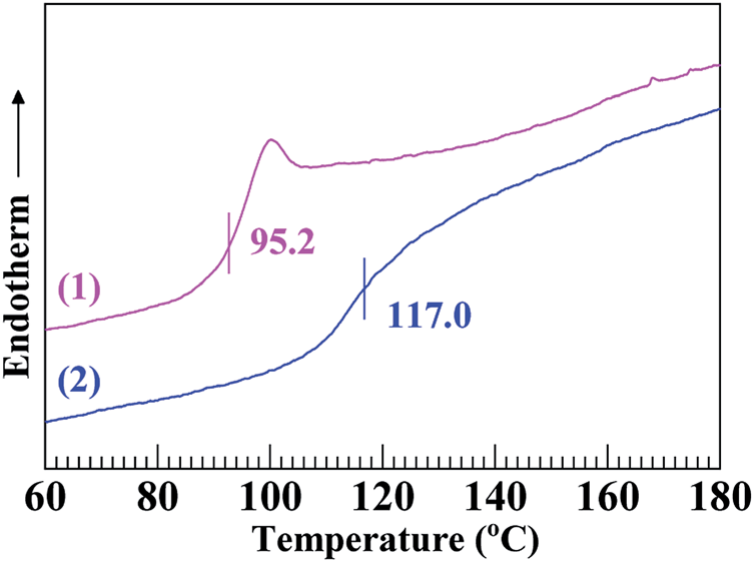
Differential scanning calorimetry (DSC) thermograms in the second heating cycle at a rate of 20 °C min^−1^ for (1) a commercial polystyrene (PS) and (2) a poly(styrene-*ran*-cinnamic acid) (SCA), *i.e.* PS copolymerised with 6.8 mol% of cinnamic acid.

Fig. 2 gives the DSC thermograms of the SCA and PS, from which their *T*_g_'s were analysed as summarised in Table 2; it is seen that the *T*_g_ (117.0 °C) of the SCA was noticeably (∼20 °C) higher than that (95.2 °C) of the PS. Also observed from Table 2 is that the HDT and VST of the SCA were both significantly enhanced until 108.0 and 118.3 °C, respectively, compared with those (87.6 and 96.0 °C) of the PS. The large increases in the *T*_g_, HDT and VST indicate that the heat resistance of the SCA was greatly improved as opposed to the PS. Again, this may be basically due to the 6.8 mol% of carboxyl groups incorporated into the SCA by copolymerisation: the steric hindrance of the pendant carboxyls limited the internal rotation of the SCA backbone chains; more remarkably, the hydrogen bond cross-linking applied by dimeric carboxyls strengthened the SCA chain-segmental interactions. Both of these led to rigidification of the SCA chains that constituted pronounced enhancement of the SCA heat resistance.

**Fig. 3:**
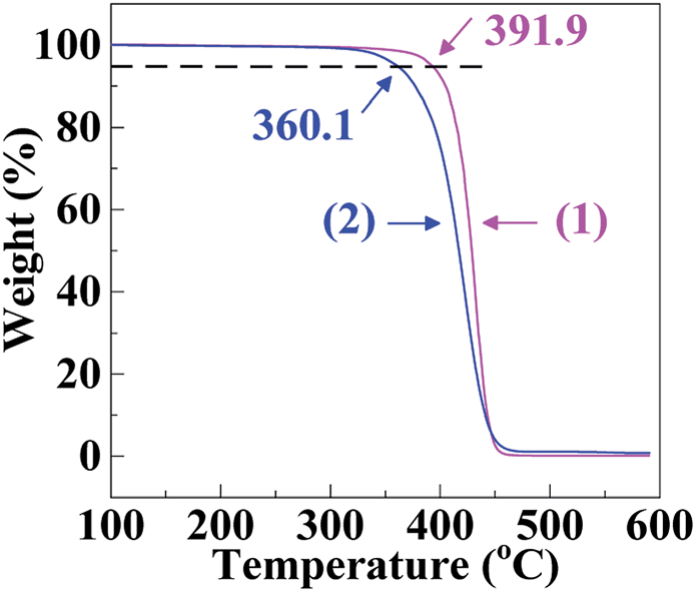
Thermogravimetric analysis (TGA) thermograms at a rate of 20 °C min^−1^ for (1) a commercial polystyrene (PS) and (2) a poly(styrene-*ran*-cinnamic acid) (SCA), *i.e.* PS copolymerised with 6.8 mol% of cinnamic acid.

It can be seen from Fig. 3 that the thermal decomposition temperature (360.1 °C) of the SCA was ∼30 °C lower than the PS (391.9 °C), the results of which are listed in Table 2. This may originate from higher chemical activity of the SCA bearing carboxyl groups, which were subject to thermal decomposition into CO_2_. Finally, as shown in Table 2, the linear thermal expansion coefficient (*α*_m_) and thermal conductivity (*λ*) of the SCA, 79.5 μm m^−1^ °C^−1^ and 0.165 W m^−1^ K^−1^ respectively, were comparable to those (78.4 μm m^−1^ °C^−1^ and 0.160 W m^−1^ K^−1^) of the PS, suggesting that the *α*_m_ and *λ* of the PS were both little changed upon the carboxylation modification.

### Melt strength of the SCA

**Fig. 4:**
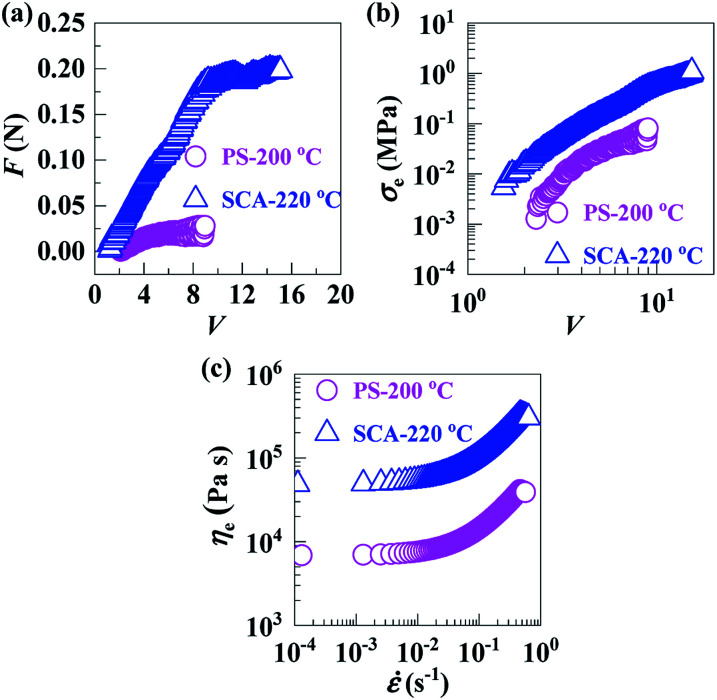
(a) Drawdown forces (*F*'s) and (b) extensional stresses (*σ*_e_'s) as functions of draw ratio (*V*) and (c) extensional viscosities (*η*_e_'s) as functions of extensional strain rate (*ε̇*), for (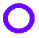) a commercial polystyrene (PS) at 200 °C and (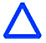) a poly(styrene-*ran*-cinnamic acid) (SCA), *i.e.* PS copolymerised with 6.8 mol% of cinnamic acid, at 220 °C.

In the molten states, with melt flow temperatures (*T*_f_) defined by HDT + 112 °C, of the PS (*T*_f_ = 200 °C) and SCA (*T*_f_ = 220 °C), the drawdown force (*F*) *vs.* draw ratio (*V*) (Fig. 4a), extensional stress (*σ*_e_) *vs. V* (Fig. 4b) and extensional viscosity (*η*_e_) *vs.* extensional strain rate (*ε̇*) (Fig. 4c) curves demonstrate that the *F*, *σ*_e_ and *η*_e_ of the SCA were all significantly larger than those of the PS, suggesting a markedly enhanced melt strength of the SCA compared with the PS. This is presumably owing to the reversible hydrogen bond cross-links as well as rigid CA inclusion; the former significantly strengthened the chain-segmental interactions, which in turn would cause a much milder decrease in the SCA melt strength with increasing temperature. In other words, compared with the PS, the SCA may exhibit a considerably broadened temperature window required for the appropriate melt strengths for stabilisation of its foaming (*i.e.* styrofoam production) operations; this constitutes a more essential sense of the SCA melt-strength improvement. As a typical measure of melt strength, the *η*_e_ (3.3 × 10^5^ Pa s) at a practical melt-processing (*e.g.* foaming) *ε̇* of 0.5 s^−1^ for the SCA is given against the PS (4.2 × 10^4^ Pa s) in Table 2.

### Processability of the SCA

Compared with the PS, the apparently sharply-reduced MFR of the SCA (0.6 *vs.* 6.9 g 10^−1^ min^−1^) shown in Table 2 does not necessarily indicate that the processing fluidity of the SCA was dramatically deteriorated at the same typical melt-processing shear rate. This is because the greatly increased viscosity of the SCA originated from not only the hydrogen bond cross-linking as well as rigid volume-fraction of CA, but, preponderantly, its much smaller shear rate (and thus shear thinning) under the same load applied during the MFR testing.

**Fig. 5:**
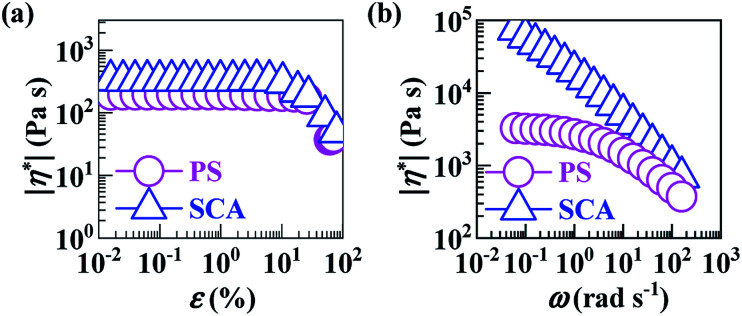
(a) Complex viscosity magnitudes (|η*|‘s) as functions of strain amplitude (*ε*) at 200 °C and 60 Hz, and (b) |η*|‘s as functions of angular frequency (*ω*) at 200 °C and an *ε* of 0.1%, for (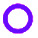) a commercial polystyrene (PS) and (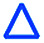) a poly(styrene-*ran*-cinnamic acid) (SCA), *i.e.* PS copolymerised with 6.8 mol% of cinnamic acid.

**Fig. 6:**
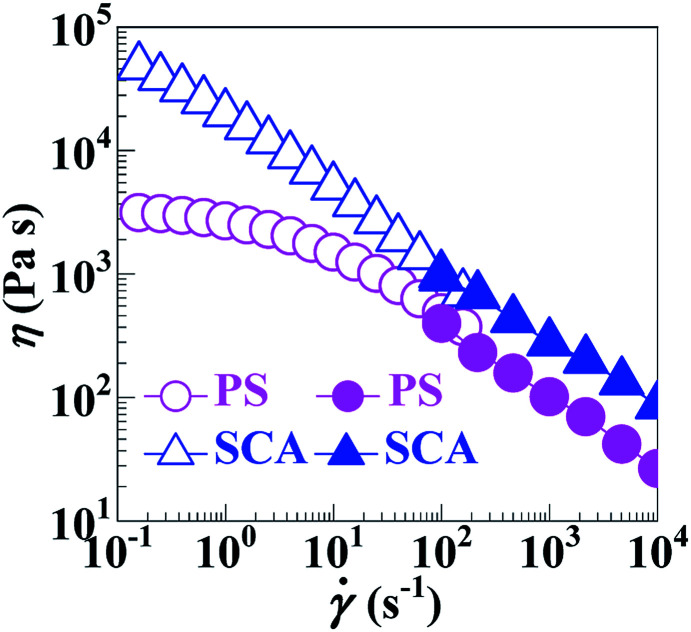
Shear-viscosity (*η*) *vs.* shear-rate (*γ̇*) flow mastercurves at 200 °C for (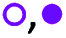) a commercial polystyrene (PS) and (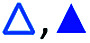) a poly(styrene-*ran*-cinnamic acid) (SCA), *i.e.* PS copolymerised with 6.8 mol% of cinnamic acid. Wherein, the data points (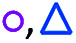) are derived from oscillatory-shear rotational rheometry and the other data points (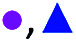) are obtained from capillary rheometry.

As can be seen from Fig. 5a, the SCA and PS were both in the linear viscoelastic region below *ε*'s of ∼5%; their |η*| *vs. ω* behaviours were therefore compared at a small *ε* of 0.1% ensuring linear viscoelasticity (Fig. 5b), which were then reduced to *η vs. γ̇* (10^−1^–10^2^ s^−1^) relationships (Fig. 6) according to the Cox–Merz rule. The capillary rheology at higher *γ̇*’s (10^2^–10^4^ s^−1^) was subsequently measured as well, and finally added to Fig. 6 to form flow mastercurves of the SCA and PS. It is seen that, over the entire range of *γ̇*’s investigated, the *η*'s of the SCA at 200 °C were higher than the PS presumably from its hydrogen bond cross-linking and rigid CA copolymerisation combined effects. Further, at low *γ̇*’s, the SCA displayed a stronger shear-thinning behaviour than the PS since its hydrogen bond cross-links were inclined to dissociate upon shearing, while, at the higher *γ̇*’s, the SCA and PS exhibited similar shear-thinning behaviours. Under these circumstances, the *η* (*e.g.* 278.9 Pa s) of the SCA at a typical melt-processing *γ̇* (*e.g.* 10^3^ s^−1^) was just a little higher relative to the PS (*e.g.* 101.7 Pa s) (Table 2), which constitutes an inheritance of the PS's excellent processability by the SCA without significant compromisation.

### Dielectric properties of the SCA

At a low frequency (*e.g.* 60 Hz), the electrical insulation (*ε* of 2.8 and tan *δ* of 3.3 × 10^−3^) of the SCA was comparable to the PS (*ε* of 2.8 and tan *δ* of 3.7 × 10^−3^); at a higher frequency (*e.g.* 1 MHz), the *ε* (2.7) of the SCA was still approximate to the PS (2.8) whereas the tan *δ* (1.6 × 10^−3^) of the SCA larger than the PS (0.6 × 10^−3^), indicating a deterioration of the SCA's electrical insulation compared with the PS (Table 2). In other words, the *ε*'s of the SCA were equivalent to the PS at both the frequencies (Table 2), which may result from the hydrogen bond cross-links of the former that essentially repressed the polarisation orientation of its carboxyls.^35,36^ However, the tan *δ* of the SCA remained close to the PS at the low frequency while became larger than the PS at the higher frequency (Table 2), the latter of which should also be associated with the weaker mobility of the SCA chain segments constrained by their hydrogen bond cross-links.

### Fire properties of the SCA

The oxygen index (19.9) of the SCA was comparable to the PS (19.7), and the density of smoke (92.5) of the SCA a bit higher than the PS (88.8) (Table 2). These results seem to discover that the fire properties of the SCA were basically in the proximity of the PS.

### Other physical properties of the SCA

The intrinsic viscosity (2.6 × 10^−2^ dL g^−1^) of the SCA was considerably lower than the PS (8.4 × 10^−2^ dL g^−1^) (Table 2), which probably arises from the hydrogen bond cross-links of the former shrinking its chain segments in radial size. The specific gravity (1.07) of the SCA was almost equal to the PS (1.05) (Table 2). And the water absorption (0.47) of the SCA was significantly increased relative to the PS (0.06) (Table 2), obviously due to its introduction of 6.8 mol% of polar, hydrophilic carboxy groups.

## Conclusions

Styrene is copolymerised *via* emulsion radical polymerisation with cinnamic acid (CA), a green compound to produce a modified polystyrene (PS), poly(styrene-*ran*-cinnamic acid) (SCA), of comparable *M̄*_w_ of ∼200 000 to commercial PS bearing 6.8 mol% of CA. Compared with commercial PS, the SCA displays significantly improved impact toughness (by 47.2%), heat resistance (glass-transition, heat deflection and Vicat softening temperatures all by ∼20 °C) and melt strength (extensional viscosity by one order of magnitude, and foaming temperature window). Regarding other properties, despite decreases in its thermal decomposition stability, electrical insulation and moisture resistance, the SCA exhibits mechanical strengths, linear thermal expansion coefficient, thermal conductivity, processability, fire properties, specific gravity, *etc*. all basically approximate to commercial PS. These overall properties of the SCA relative to commercial PS, from a chain topological viewpoint, are all attributed to its rigid volume-fraction of CA and/or hydrogen bond cross-linking from dimerisation of CA carboxyls. As a modified version of PS, the SCA demonstrates both-improved, balanced impact toughness and heat resistance superior to commercial HIPS, SMA, SAN, SMMA and ABS, as well as enhanced melt strength, which potentially extend its styrofoam and commodity applications.

## Conflicts of interest

There are no conflicts of interest to declare.

## Supplementary Material
